# Association of Preconception Blood Pressure with the Risk of Anemia in Children under Five Years of Age: A Large Longitudinal Chinese Birth Cohort

**DOI:** 10.3390/nu14132640

**Published:** 2022-06-26

**Authors:** Hang An, Huiting Chen, Zhiwen Li, Le Zhang, Yali Zhang, Jianmeng Liu, Rongwei Ye, Nan Li

**Affiliations:** 1 Ministry of Health Key Laboratory of Reproductive Health, Institute of Reproductive and Child Health, Peking University Health Science Center, Beijing 100191, China; anhang@bjmu.edu.cn (H.A.); htchen1999@163.com (H.C.); lizw@bjmu.edu.cn (Z.L.); zhangle@bjmu.edu.cn (L.Z.); zhangyl@bjmu.edu.cn (Y.Z.); liujm@pku.edu.cn (J.L.); 2 Department of Epidemiology and Biostatistics, School of Public Health, Peking University Health Science Center, Beijing 100191, China

**Keywords:** blood pressure, Chinese women, anemia, children, hypertension

## Abstract

Hypertension during pregnancy may increase the risk of anemia in the offspring. However, few studies have investigated the effects of elevated blood pressure during the preconception period on childhood anemia. This large population-based birth cohort study was performed to determine whether abnormal preconception blood pressure has long-term consequences for childhood health. Data were obtained from the China–US Collaborative Project for Neural Tube Defect Prevention. The study consisted of 40,638 women with singleton live births who were registered in a monitoring system before pregnancy in southern China during the period 1993–1996. Children were assessed by hemoglobin measurement at approximately 53 months of age. The incidences of childhood anemia were 19.80% in the hypertension group and 16.07% in the non-hypertension group. Compared with the non-hypertension group, the hypertension group had an increased risk of childhood anemia (adjusted risk ratio (RR): 1.25; 95% confidence interval (CI): 1.11–1.41). After categorization according to blood pressure, combined systolic and diastolic hypertension was associated with a significantly increased risk of childhood anemia, compared with normotension (adjusted RR: 1.37; 95% CI: 1.16–1.63). Compared with women who had normal blood pressure, the adjusted RRs for childhood anemia were 1.20 (95% CI: 1.13–1.28), 1.26 (95% CI: 1.08–1.47), and 1.38 (95% CI: 1.14–1.67) among women with prehypertension, stage-1 hypertension, and stage-2 hypertension, respectively. Our results suggest a linear association between prepregnancy hypertension and the risk of childhood anemia in the Chinese population. Interventions targeting preconception blood pressure may have a positive effect on childhood health.

## 1. Introduction

Anemia is a global public health problem that disproportionately affects children in developing countries. The estimated global prevalence of anemia in children under 5 years of age is 43% [1], which is important because anemia is associated with childhood morbidity and mortality, as well as the potential for long-term health effects [2,3,4]. Among the risk modifiers of childhood anemia, iron deficiency is the most common cause [5,6]. Although diet and medication during pregnancy can have an effect on iron absorption in children, programs involving supplementation with iron or folic acid have only demonstrated moderate success. Research concerning the factors that influence iron status (e.g., nutrition status and lifestyle factors) has historically focused on the period during pregnancy [7,8].

The placenta has a pivotal role in iron transport and storage to buffer both the mother and fetus from large shifts in nutrient availability [9]. Hypertensive disorders of pregnancy (HDP) are reportedly associated with poor placental function [10,11]. Some studies have suggested that HDP is associated with an increased risk of childhood anemia [12,13,14]. Despite the biological rationale, there have been few studies regarding the association between preconception blood pressure and childhood anemia. From a public health perspective, understanding the impact of preconception blood pressure is of particular interest because it is generally easy to intervene before pregnancy; such interventions may have greater impacts on gestational outcomes. Therefore, we conducted a large cohort study to examine the possible relationship between preconception blood pressure and childhood anemia.

Several studies have suggested that preconception blood pressure is associated with adverse pregnancy outcomes, especially pregnancy loss and preterm birth [15,16,17], potentially through placental vascular insufficiency, which is a common source of iron deficiency. However, few studies have compared the influence among blood pressure categories, possibly because of limited sample sizes. Prior research has indicated that diastolic blood pressure (DBP) may strongly contribute to proximal pathophysiological processes [16], and these effects might differ among stages of hypertension [15]. Therefore, large cohort studies are needed to compare the effects of preconception blood pressure level to improve our understanding of the mechanisms underlying each subtype. Here, we used data from a large birth cohort study to evaluate the association of preconception blood pressure with childhood anemia; we aimed to identify both overall and subgroup effects.

## 2. Materials and Methods

### 2.1. Study Design

**Figure 1:**
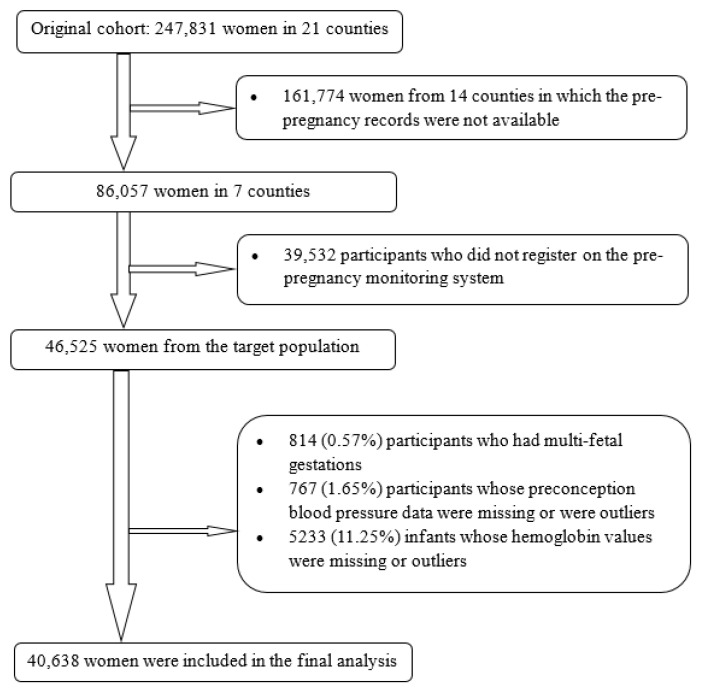
Flowchart of participants.

The original study was described previously [18,19]. Briefly, the Chinese Ministry of Health conducted a public health campaign to prevent neural tube defects. This campaign involved a large prospective cohort study to investigate the effects of preconception use of folic acid on neural tube defects, as well as child growth and development. Beginning in 1993, the campaign was implemented in 21 counties in two southern provinces (Zhejiang and Jiangsu) and one northern province (Hebei). All women in the study who were preparing for marriage or who became pregnant were registered in a perinatal health care surveillance system; records were kept regarding their prenatal care and demographic information. This surveillance system followed the registered women until the conclusion of their pregnancies and recorded all births at or after 20 gestational weeks, including live births and stillbirths. Furthermore, all children delivered by the registered women during the study period were assessed by physical examination. The original cohort included 247,831 women; for this analysis, we selected seven counties in two neighboring southern provinces (Jiangsu Province and Zhejiang Province), which had detailed records of preconception examinations as well as gestational weeks, enabling us to examine the effect of preconception blood pressure and risk of childhood anemia and to differentiate the effects according to hypertension categories. Of 86,057 women from the selected counties, we chose 46,525 women who registered on the health examination system before pregnancy as our target population. Of these women, we excluded 814 (0.57%) with multifetal gestations, 767 (1.65%) with missing preconception blood pressure information or whose values were outliers, and 5233 (11.25%) with infants whose hemoglobin levels were missing or whose values were outliers (hemoglobin concentration <60 g/L or >200 g/L). After these exclusions, 40,638 mother–child pairs (87.35% of the target population) were included in the analysis (Figure 1).

The project was approved by the institutional review boards of the US Centers for Disease Control and Prevention and Peking University Health Science Center. The institutional review boards determined that these secondary analyses of previously collected data were exempt from the requirement for informed consent.

### 2.2. Exposure and Covariates

Blood pressure was assessed by trained health care workers at the time of registration, approximately three months prior to conception. The blood pressure cuff bladder was adjusted to the appropriate size based on arm circumference. Blood pressure in the right arm was measured with a mercury sphygmomanometer; it was recorded on ≥2 consecutive occasions, with an interval of ≥6 h. Hypertension was defined as systolic blood pressure (SBP) ≥ 140 mmHg and/or DBP ≥ 90 mmHg. To investigate the associations of different types of hypertension with childhood anemia, blood pressure was further categorized in accordance with the classifications of the World Health Organization and International Society of Hypertension Guidelines on Hypertension Management [20], as follows: normotension (SBP < 140 mmHg and DBP < 90 mmHg), isolated systolic hypertension (SBP ≥ 140 mmHg and DBP < 90 mmHg), isolated diastolic hypertension (SBP < 140 mmHg and DBP ≥ 90 mmHg), and combined hypertension (SBP ≥ 140 mmHg and DBP ≥ 90 mmHg). Based on the 7th Report of the Joint National Committee on Prevention, Detection, Evaluation, and Treatment of High Blood Pressure [21], individuals were divided into the following four categories: normal blood pressure (SBP < 120 mmHg and DBP < 80 mmHg), prehypertension (SBP 120–139 mmHg and/or DBP 80–89 mmHg), stage-1 hypertension (SBP 140–159 mmHg and/or DBP 90–99 mmHg), and stage-2 hypertension (SBP ≥ 160 mmHg and/or DBP ≥ 100 mmHg). Isolated systolic blood pressure and isolated diastolic blood pressure were also transformed by per 1-SD (standard deviation) increment in respective blood pressure levels.

At enrollment, all women were issued a perinatal health care booklet designed specifically for this project. Basic maternal characteristics were first input into the booklet by trained doctors, then entered into computers by trained staff from the maternal and child health hospitals with a telephone modem-based data transfer system; the data were subsequently transferred to the Peking University Project Center. The information included age (years), ethnicity (Han or other), education (high school or higher, junior high school, elementary school or lower, or unknown), occupation (farmer or other), parity (primigravida or multigravida), and periconceptional folic acid consumption status (yes or no). The prepregnancy body mass index (BMI, kg/m^2^) was calculated from the assessed weight and height. Women with anemia during pregnancy were diagnosed according to the threshold of hemoglobin <110 g/L. Feeding practices (exclusive breastfeeding or not) were self-reported during the home visit at 42 days after birth. These records were collected using a consistently structured questionnaire and entered into the booklet, then entered into computers by trained staff in hospitals, and finally transferred to the Peking University Project Center.

### 2.3. Hemoglobin Measurement

Hemoglobin levels in children were measured by trained doctors using finger-prick blood samples in accordance with standard procedures. A step-by-step instruction leaflet was pasted on the wall of each examination room to ensure compliance. To minimize testing bias, the rooms were equipped with heating devices in winter to maintain a temperature of >18 °C. All participating hospitals conducted measurements according to a standard cyanmethemoglobin method with two commonly used devices: a visible spectrophotometer and a hemoglobinometer. They were provided standard hemoglobin solutions (50, 100, 150, and 200 g/L) and instructions for calibrating the hemoglobinometer and preparing the standard curve for the 721 visible spectrophotometers [22]. Childhood anemia was defined as a hemoglobin concentration <110 g/L for children aged <60 months, in accordance with the recommendations of the World Health Organization [23].

### 2.4. Statistical Analysis

We compared women with and without preconception hypertension in terms of age, BMI, parity, ethnicity, folic acid use, education, occupation, maternal anemia during pregnancy, feeding practices, and child’s age at the follow-up visit. Student’s *t*-test was used for analyses of quantitative variables; the χ^2^ test was used for analyses of categorical variables. Logistic regression analyses were used to estimate risk ratios (RRs) of childhood anemia with maternal preconception hypertension after adjusting for the following confounders: maternal age (continuous), BMI (continuous), parity, ethnicity, folic acid use, education, anemia during pregnancy, occupation, feeding practices, and age at the follow-up visit. All data were analyzed using SPSS for Windows software (ver. 20.0; SPSS Inc., Chicago, IL, USA).

## 3. Results

In total, 40,638 mother–child pairs were included in this study; 1808 (4.45%) of the women had preconception hypertension. Table 1 shows the characteristics of women with and without preconception hypertension. Older women with higher BMI, multiparous status, factory employment, anemia during pregnancy and children with younger age at follow-up visit were more likely to have preconception hypertension.

The incidences of childhood anemia were 19.80% and 16.07% among children of mothers with and without preconception hypertension, respectively. Maternal preconception hypertension was associated with an increased risk of childhood anemia. The association remained after adjustment for confounders (adjusted RR = 1.25, 95% CI (confidence interval): 1.11–1.41). The preconception hypertension group had a lower hemoglobin level than did the non-hypertension group (116.78 g/L vs. 118.34 g/L, respectively, *p* < 0.001). However, preconception hypertension showed a borderline significant association with reduction of the hemoglobin level after adjustment for multiple covariates (Table 2).

The incidences of childhood anemia and adjusted RRs across blood pressure categories are shown in Table 3. Compared with normotensive women, the children of women with isolated systolic hypertension or isolated diastolic hypertension prior to pregnancy did not exhibit a significantly increased risk of childhood anemia, whereas combined hypertension was positively associated with childhood anemia (adjusted RR = 1.37, 95% CI: 1.16–1.63). An increased risk of childhood anemia was observed in mother–child pairs with any degree of preconception hypertension (prehypertension: adjusted RR = 1.20, 95% CI: 1.13–1.28; stage-1 hypertension: adjusted RR = 1.26, 95% CI: 1.08–1.47; stage-2 hypertension: adjusted RR = 1.38, 95% CI: 1.14–1.67). Linear-by-linear association analysis also demonstrated a significant trend. Furthermore, we found that preconception SBP and DBP (per 1-standard deviation increase) were associated with 9% and 11% increased risks of childhood anemia, respectively.

## 4. Discussion

The results of this large longitudinal birth cohort showed that preconception hypertension was associated with an increased risk of childhood anemia in children under 5 years of age. After categorization according to blood pressure, both increased SBP and DBP demonstrated adverse effects on childhood anemia. All degrees of preconception hypertension (i.e., prehypertension, stage-1 hypertension, and stage-2 hypertension) were associated with an increased risk of childhood anemia; the risk increased as the hypertension severity increased. These results highlight the importance of blood pressure assessment during the prepregnancy period for the detection and prevention of childhood anemia.

Our hypothesis regarding an association of preconception blood pressure with childhood anemia was based on the influence of HDP on childhood anemia. Previous studies suggest that HDP affects iron storage in children; this condition may be positively associated with the risk of anemia [12,24,25]. In Kim et al. [14], the authors reported that neonates of mothers with HDP had lower total body iron stores than did neonates of mothers without HDP. This association was observed in appropriate for gestational age infants [median (interquartile range): with HDP, 183.7 (154.3–230.2) ng/mL vs. without HDP, 206.1 (172.6–244.6) ng/mL] and smaller amounts for gestational age infants [163.8 (121.5–185.4) ng/mL vs. 194.61 (163.4–217.8) ng/mL]. The serum ferritin levels of the neonates showed similar results. Another cohort study conducted in the Netherlands [13] also suggested that HDP is associated with decreased iron transport, leading to negative effects on iron status in children. However, these studies did not consider the effects of preconception blood pressure level. The preconception period is a critical time for interventions that can positively influence health in both mothers and children. Therefore, we speculated that preconception high blood pressure leads to childhood anemia.

Our results suggested that incrementally higher preconceptions of SBP and DBP contribute to the risk of childhood anemia. These findings were similar to previous research concerning HDP. A cohort study derived from the EAGeR (Effects of Aspirin on Gestation and Reproduction) trial [26] reported that both higher preconception SBP and DBP were associated with an increased risk of HDP (adjusted RR: 1.05, 95% CI: 1.00–1.10, per 2 mmHg diastolic blood pressure; adjusted RR: 1.07, 95% CI: 1.00–1.13, per 2 mmHg mean arterial pressure). We presumed that elevated preconception blood pressure indicates an increased risk of higher blood pressure during pregnancy and may be associated with infant health status. The mechanisms underlying this observation may be related to the impaired placental function that arises from elevated blood pressure [11,27]. Endothelial dysfunction and maternal vascular malperfusion have been identified as characteristics of elevated preconception blood pressure [28,29]. Poor placental status reduces the capacity to store iron in resident reticuloendothelial cells [30]; it may also restrict iron transport during pregnancy. Some studies have shown that children born with lower fetal iron loading have a greater risk of postnatal iron deficiency [31,32]. Therefore, elevated blood pressure before pregnancy may result in limited iron storage in children, leading to anemia.

An important finding in this study was that, compared with the children of mothers with normal blood pressure, the children of mothers with prehypertension (SBP 120–139 mmHg and/or DBP 80–89 mmHg) were more likely to be anemic. This result strengthens the growing body of evidence that suggests prehypertension may lead to increased rates of adverse perinatal outcomes [16,17]. However, previous studies focused mainly on short-term adverse pregnancy outcomes, while our results suggested that the effects of maternal prehypertension on infants persist until the age of 53 months. Interventions to manage prehypertension among women of childbearing age may reduce the risks of adverse outcomes in both women and their children, considering that 41.3% of the adult population in China reportedly exhibits prehypertension based on the criteria used in our analysis [33]. Because prehypertension is not a disease category, there are currently no drug therapy recommendations for blood pressure control among women with prehypertension. Lifestyle modifications, such as weight reduction [34], regular physical activity [35], and adherence to the Dietary Attempts to Stop Hypertension (DASH) diet [36], have been reported to aid in blood pressure control. Additional research is needed to determine whether maternal clinical treatment or lifestyle modifications can prevent childhood anemia.

This study had several strengths. First, it used data from a population-based longitudinal birth cohort study, which enabled avoidance of selection bias and recall bias. In addition, the perinatal health care surveillance system guaranteed follow-up data concerning pregnancy outcomes and childhood health. Data collection and diagnoses of both hypertension and childhood anemia were conducted by trained health workers or doctors using uniform procedures. Second, >80% of children were finally matched with their mothers, which generated a sufficient sample size to detect both the effects of overall and different degrees of preconception hypertension on childhood anemia. Moreover, most of the women in this study were of Han ethnicity, which ensured a homogeneous genetic background. The women all lived in similar regions under similar conditions, which facilitated comparisons between groups. Finally, we measured hemoglobin concentrations in children at approximately 53 months of age, which enabled exploration of the long-term effects of maternal preconception hypertension.

This study also had some limitations. First, although the analysis was able to control for the effects of many factors, the use of existing data may have permitted residual confounding effects of maternal smoking and alcohol use. However, women—especially women preparing to become pregnant—are less likely to smoke or drink alcohol in rural China. Maternal covariates information on worm infestation, maternal hygiene, and other childhood characteristics were also unavailable in this study. Further studies are needed to collect this information and test its possible effects. Second, indicators of iron storage, such as serum ferritin and transferrin, were not measured in this study; this led to difficulties in identifying types of childhood anemia. Further studies with more sensitive markers are required to investigate possible mechanisms underlying the observed associations. Third, childhood anemia was only diagnosed during one visit at the age of 53 months. Repeated measurements of hemoglobin are needed to observe the effects of hypertension at different time points. Fourth, the original study was conducted in two southern provinces and one northern province. However, the northern province was excluded from the final analysis because relevant clinical records were unavailable for this study. Fifth, our study population was largely of Han ethnicity; further studies are therefore required to confirm the generalizability of the findings across ethnicities and regions. Finally, although our findings indicated an association between preconception hypertension and an increased risk of childhood anemia, we could not establish causality. Further studies are needed to explore the mechanisms underlying the observed associations.

## 5. Conclusions

In conclusion, our findings suggest that higher maternal preconception blood pressure level is associated with an increased risk of childhood anemia. Further research in diverse populations is required to determine whether management of preconception hypertension in women can prevent anemia in their children.

## Figures and Tables

**Table 1:** Baseline characteristics of women according to preconception hypertension in China.

Characteristics	Hypertension Group (*n* = 1808)		Non-Hypertension Group (*n* = 38,830)		*p*
	*n*	%	*n*	%	
Age (years, mean [SD])	25.00 (2.52)		24.67 (2.25)		<0.001
Body mass index (kg/m^2^, mean [SD])	20.55 (2.21)		20.38 (2.14)		0.002
Primiparous	1643	90.87	36 055	92.85	0.002
Han ethnicity	1798	99.45	38 621	99.46	0.869
Folic acid use	1431	79.15	30 619	78.85	0.789
Education					0.072
High school or higher	156	8.63	3521	9.07	
Junior high school	1039	57.47	23 137	59.59	
Elementary school or lower, or unknown	613	33.90	12 172	31.35	
Occupation					<0.001
Farmer	1052	58.19	25 687	66.15	
Factory worker	611	33.79	8759	22.56	
Other or known	145	8.02	4384	11.29	
Anemia during pregnancy	1308	72.35	24330	62.66	<0.001
Exclusive breastfeeding	1638	90.60	34 845	89.74	0.250
Child					
Age at follow-up visit (months, mean [SD])	52.23 (7.90)		53.13 (7.93)		<0.001
SD, standard deviation.					

**Table 2:** Associations of childhood anemia incidence and hemoglobin level (g/L) with preconception hypertension in China.

	Hypertension Group (*n* = 1808)		Non-Hypertension Group (*n* = 38,830)				
	No. of Cases	Incidence (%)	No. of Cases	Incidence (%)	Crude RR (95% CI)	Adjusted RR (95% CI) *	Adjusted RR (95% CI) ^†^
Childhood anemia	358	19.80	6241	16.07	1.29 (1.15–1.45)	1.26 (1.12–1.42)	1.25 (1.11–1.41)
Hemoglobin (g/L, mean [SD])	116.78 (9.78)		118.34 (10.14)		1.56 (1.08–2.04)	1.52 (1.04–2.00)	1.46 (0.98–1.94)

No., number; RR, risk ratio; CI, confidence interval; SD, standard deviation. * Adjusted for maternal age (continuous), body mass index (BMI) (continuous), education, occupation, folic acid use, ethnicity, and parity. ^†^ Adjusted for maternal age (continuous), BMI (continuous), education, occupation, folic acid use, ethnicity, parity, feeding practices, anemia during pregnancy, and age at the follow-up visit.

**Table 3:** Childhood anemia incidence and risk according to maternal blood pressure category in China.

Blood Pressure Category	No. of Participants	Childhood Anemia Incidence (%)	Crude RR (95% CI)	Adjusted RR (95% CI) *	Adjusted RR (95% CI) ^†^
Classification 1					
Normotension	38 830	16.07	1	1	1
Isolated systolic hypertension	810	18.15	1.16 (0.97–1.39)	1.12 (0.94–1.35)	1.12 (0.94–1.35)
Isolated diastolic hypertension	149	19.46	1.26 (0.84–1.90)	1.27 (0.84–1.91)	1.25 (0.83–1.88)
Combined hypertension ^‡^	849	21.44	1.43 (1.21–1.68)	1.40 (1.19–1.66)	1.37 (1.16–1.63)
Classification 2					
Normal blood pressure	30 337	15.47	1	1	1
Prehypertension	8493	18.22	1.22 (1.14–1.30)	1.20 (1.12–1.27)	1.20 (1.13–1.28)
Stage-1 hypertension	1142	19.18	1.30 (1.12–1.51)	1.27 (1.09–1.47)	1.26 (1.08–1.47)
Stage-2 hypertension ^§^	666	20.87	1.44 (1.19–1.74)	1.41 (1.17–1.71)	1.38 (1.14–1.67)
Classification 3 **					
Isolated systolic per 1-SD increase	40 638	16.23	1.10 (1.08–1.13)	1.10 (1.07–1.12)	1.09 (1.07–1.12)
Isolated diastolic per 1-SD increase	40 638	16.23	1.12 (1.10–1.15)	1.11 (1.08–1.14)	1.11 (1.08–1.14)

No., number; RR, risk ratio; CI, confidence interval; SD, standard deviation. * Adjusted for: maternal age (continuous), body mass index (BMI) (continuous), education, occupation, folic acid use, ethnicity, and parity. ^†^ Adjusted for maternal age (continuous), BMI (continuous), education, occupation, folic acid use, ethnicity, parity, feeding practices, anemia during pregnancy, and age at the follow-up visit. ^‡^ Test for trend (*p* < 0.001): comparison of hypertension classification 1 between groups with and without childhood anemia. ^§^ Test for trend (*p* < 0.001): comparison of hypertension classification 2 between groups with and without childhood anemia. ** The isolated systolic per 1-SD and isolated diastolic per 1-SD were 11.69 mmHg and 8.71 mmHg, respectively.

## Data Availability

The data are available in the main text, or can be obtained by contacting the corresponding author (N.L.).

## References

[1] Stevens G.A., Finucane M.M., De-Regil L.M., Paciorek C.J., Flaxman S.R., Branca F., Peña-Rosas J.P., Bhutta Z.A., Ezzati M. (2013). Global, regional, and national trends in haemoglobin concentration and prevalence of total and severe anaemia in children and pregnant and non-pregnant women for 1995-2011: A systematic analysis of population-representative data. Lancet Glob. Health.

[2] Grantham-McGregor S., Ani C. (2001). A review of studies on the effect of iron deficiency on cognitive development in children. J. Nutr..

[3] Balarajan Y., Ramakrishnan U., Ozaltin E., Shankar A.H., Subramanian S.V. (2011). Anaemia in low-income and middle-income countries. Lancet.

[4] Scott S.P., Chen-Edinboro L.P., Caulfield L.E., Murray-Kolb L.E. (2014). The impact of anemia on child mortality: An updated review. Nutrients.

[5] Ezzati M.L.A., Rodgers A., Murray C.J. (2005). Comparative Quantification of Health Risks: Global and Regional Burden of Disease Attributable to Selected Major Risk Factors.

[6] Cameron B.M., Neufeld L.M. (2011). Estimating the prevalence of iron deficiency in the first two years of life: Technical and measurement issues. Nutr. Rev..

[7] Kumera G., Haile K., Abebe N., Marie T., Eshete T. (2018). Anemia and its association with coffee consumption and hookworm infection among pregnant women attending antenatal care at Debre Markos Referral Hospital, Northwest Ethiopia. PLoS ONE.

[8] Aşcı Ö., Rathfisch G. (2016). Effect of lifestyle interventions of pregnant women on their dietary habits, lifestyle behaviors, and weight gain: A randomized controlled trial. J. Health Popul. Nutr..

[9] McArdle H.J., Gambling L., Kennedy C. (2014). Iron deficiency during pregnancy: The consequences for placental function and fetal outcome. Proc. Nutr. Soc..

[10] Naderi S., Tsai S.A., Khandelwal A. (2017). Hypertensive Disorders of Pregnancy. Curr. Atheroscler. Rep..

[11] Rana S., Lemoine E., Granger J.P., Karumanchi S.A. (2019). Preeclampsia: Pathophysiology, Challenges, and Perspectives. Circ. Res..

[12] Chockalingam U.M., Murphy E., Ophoven J.C., Weisdorf S.A., Georgieff M.K. (1987). Cord transferrin and ferritin values in newborn infants at risk for prenatal uteroplacental insufficiency and chronic hypoxia. J. Pediatr..

[13] Uijterschout L., Vloemans J., Rövekamp-Abels L., Feitsma H., van Goudoever J.B., Brus F. (2014). The influences of factors associated with decreased iron supply to the fetus during pregnancy on iron status in healthy children aged 0.5 to 3 years. J. Perinatol..

[14] Kim H.A., Park S.H., Lee E.J. (2019). Iron status in small for gestational age and appropriate for gestational age infants at birth. Korean J. Pediatr..

[15] Yang Y., He Y., Li Q., Wang Y., Peng Z., Xu J., Ma X. (2015). Preconception blood pressure and risk of preterm birth: A large historical cohort study in a Chinese rural population. Fertil. Steril..

[16] Nobles C.J., Mendola P., Mumford S.L., Naimi A.I., Yeung E.H., Kim K., Park H., Wilcox B., Silver R.M., Perkins N.J. (2018). Preconception Blood Pressure Levels and Reproductive Outcomes in a Prospective Cohort of Women Attempting Pregnancy. Hypertension.

[17] Greenberg V.R., Silasi M., Lundsberg L.S., Culhane J.F., Reddy U.M., Partridge C., Lipkind H.S. (2021). Perinatal outcomes in women with elevated blood pressure and stage 1 hypertension. Am. J. Obstet. Gynecol..

[18] Berry R.J., Li Z., Erickson J.D., Li S., Moore C.A., Wang H., Mulinare J., Zhao P., Wong L.Y., Gindler J. (1999). Prevention of neural-tube defects with folic acid in China. China-U.S. Collaborative Project for Neural Tube Defect Prevention. N. Engl. J. Med..

[19] Gindler J., Liu J., Berry R., Li Z., Correa A., Wang H., Wang Y. (2001). Growth of children whose mothers took folic acid during early pregnancy-Sino-US NTD Project. Paediatr. Perinat. Epidemiol..

[20] Whitworth J.A., Chalmers J. (2004). World health organisation-international society of hypertension (WHO/ISH) hypertension guidelines. Clin. Exp. Hypertens..

[21] Chobanian A.V., Bakris G.L., Black H.R., Cushman W.C., Green L.A., Izzo J.L., Jones D.W., Materson B.J., Oparil S., Wright J.T. (2003). The Seventh Report of the Joint National Committee on Prevention, Detection, Evaluation, and Treatment of High Blood Pressure: The JNC 7 report. JAMA.

[22] Li H.T., Trasande L., Zhu L.P., Ye R.W., Zhou Y.B., Liu J.M. (2015). Association of cesarean delivery with anemia in infants and children in 2 large longitudinal Chinese birth cohorts. Am. J. Clin. Nutr..

[23] WHO WHO Recommendations for the Prevention and Treatment of Postpartum Haemorrhage. http://www.who.int/reproductivehealth/publications/maternal_perinatal_health/9789241548502/en/.

[24] Siddappa A.M., Rao R., Long J.D., Widness J.A., Georgieff M.K. (2007). The assessment of newborn iron stores at birth: A review of the literature and standards for ferritin concentrations. Neonatology.

[25] Georgieff M.K. (2008). The role of iron in neurodevelopment: Fetal iron deficiency and the developing hippocampus. Biochem. Soc. Trans..

[26] Nobles C.J., Mendola P., Mumford S.L., Silver R.M., Kim K., Andriessen V.C., Connell M., Sjaarda L., Perkins N.J., Schisterman E.F. (2020). Preconception Blood Pressure and Its Change Into Early Pregnancy: Early Risk Factors for Preeclampsia and Gestational Hypertension. Hypertension.

[27] Rao R., Georgieff M.K. (2007). Iron in fetal and neonatal nutrition. Semin. Fetal Neonatal Med..

[28] Everett T.R., Lees C.C. (2012). Beyond the placental bed: Placental and systemic determinants of the uterine artery Doppler waveform. Placenta.

[29] Braunthal S., Brateanu A. (2019). Hypertension in pregnancy: Pathophysiology and treatment. SAGE Open Med..

[30] Georgieff M.K. (2020). Iron deficiency in pregnancy. Am. J. Obstet. Gynecol..

[31] Georgieff M.K., Wewerka S.W., Nelson C.A., Deregnier R.A. (2002). Iron status at 9 months of infants with low iron stores at birth. J. Pediatr..

[32] Shao J., Richards B., Kaciroti N., Zhu B., Clark K.M., Lozoff B. (2021). Contribution of iron status at birth to infant iron status at 9 months: Data from a prospective maternal-infant birth cohort in China. Eur. J. Clin. Nutr..

[33] Wang Z., Chen Z., Zhang L., Wang X., Hao G., Zhang Z., Shao L., Tian Y., Dong Y., Zheng C. (2018). Status of Hypertension in China: Results from the China Hypertension Survey, 2012–2015. Circulation.

[34] Landsberg L., Aronne L.J., Beilin L.J., Burke V., Igel L.I., Lloyd-Jones D., Sowers J. (2013). Obesity-related hypertension: Pathogenesis, cardiovascular risk, and treatment: A position paper of The Obesity Society and the American Society of Hypertension. J. Clin. Hypertens..

[35] Cornelissen V.A., Buys R., Smart N.A. (2013). Endurance exercise beneficially affects ambulatory blood pressure: A systematic review and meta-analysis. J. Hypertens..

[36] Chiavaroli L., Viguiliouk E., Nishi S.K., Blanco Mejia S., Rahelić D., Kahleová H., Salas-Salvadó J., Kendall C.W., Sievenpiper J.L. (2019). DASH Dietary Pattern and Cardiometabolic Outcomes: An Umbrella Review of Systematic Reviews and Meta-Analyses. Nutrients.

